# GOChase-II: correcting semantic inconsistencies from Gene Ontology-based annotations for gene products

**DOI:** 10.1186/1471-2105-12-S1-S40

**Published:** 2011-02-15

**Authors:** Yu Rang Park, Jihun Kim, Hye Won Lee, Young Jo Yoon, Ju Han Kim

**Affiliations:** 1 Seoul National University Biomedical Informatics (SNUBI), Div. of Biomedical Informatics, Seoul National University College of medicine, Seoul 110799, Korea; 2 Department of Molecular Genetics and Microbiology, University of Florida, Gainesville, USA; 3 Systems Biomedical Informatics Research Center, Seoul National University, Seoul 110799, Korea

## Abstract

**Background:**

The Gene Ontology (GO) provides a controlled vocabulary for describing genes and gene products. In spite of the undoubted importance of GO, several drawbacks associated with GO and GO-based annotations have been introduced. We identified three types of semantic inconsistencies in GO-based annotations; semantically redundant, biological-domain inconsistent and taxonomy inconsistent annotations.

**Methods:**

To determine the semantic inconsistencies in GO annotation, we used the hierarchical structure of GO graph and tree structure of NCBI taxonomy. Twenty seven biological databases were collected for finding semantic inconsistent annotation.

**Results:**

The distributions and possible causes of the semantic inconsistencies were investigated using twenty seven biological databases with GO-based annotations. We found that some evidence codes of annotation were associated with the inconsistencies. The numbers of gene products and species in a database that are related to the complexity of database management are also in correlation with the inconsistencies. Consequently, numerous annotation errors arise and are propagated throughout biological databases and GO-based high-level analyses. GOChase-II is developed to detect and correct both syntactic and semantic errors in GO-based annotations.

**Conclusions:**

We identified some inconsistencies in GO-based annotation and provided software, GOChase-II, for correcting these semantic inconsistencies in addition to the previous corrections for the syntactic errors by GOChase-I.

## Background

The Gene Ontology (GO) project started to provide semantic standards for the annotation of molecular attributes of genes and gene products [1]. The Gene Ontology is a controlled vocabulary for describing genes and gene products in terms of their associated biological processes, cellular components and molecular functions. The structural foundation of GO is formally a Directed Acyclic Graph (DAG) wherein the terms are equivalent to the nodes and the relationships to the edges of the graph [2].

GO has grown enormously. The number of organism groups participating in the GO Consortium has grown every quarter year from the initial three to roughly two dozen [3]. A lot of biological databases use GO to annotate the molecular attributes of genes and gene products [4,5]. GO-based analysis of microarray and mass spectrometry data have been successfully realized [3]. Recently, new generation of tools based-on GO have been developed, aiming to enhance biological knowledge such as protein structure classifying [6], gene-phenotype association predicting [7] and gene network building [8]. More details are available at GO website (http://www.geneontology.org/GO.tools.shtml). Unified Medical Language System (UMLS) metathesaurus has been integrated with GO to expand UMLS into the biological domain [9].

In spite of the undoubted importance of GO, several drawbacks associated with GO and GO-based annotations have been introduced. Masseroli correctly pointed out the structural and semantic problems of GO such as metonymy, species-specific terms and multiple paths [10]. Dolan et al. evaluated the reliability of GO-based annotations [11]. Poor inter-annotator reliability of GO-based annotations for human-mouse orthologous gene pairs was reported between two gene-annotation groups, MGI and GOA. Park et al. identified syntactic errors caused by the two GO-update operations, ‘new obsoletions’ and ‘new term merges’, used in the course of GO version change [12]. They introduced GOChase to detect and correct the syntactic errors and error propagations in GO-based annotations (http://www.snubi.org/software/GOChase/).

In the present study, we further identified semantic error types in GO-based annotations; redundant, biological-domain-inconsistent and taxonomy inconsistent annotation.

The first type is “redundant annotation.” When a gene is annotated to a GO term, for instance, according to the current GO annotation paradigm, it is considered to be implicitly annotated to all parents of the term. Assigning both parent and child terms to the same gene is regarded as “redundant annotation.” In some cases, if parent and child term was annotated in specific gene product using different evidence code, these annotations hard to say completely redundant. For example, an experiment may provide enough evidence to annotate to a parent, but not to any specific child, whereas a more specific annotation may be predicted by sequence comparison or other computation. In such cases both annotation would be retained, the parent because of its experiment support and the child for specificity. So we analyze the redundant annotation to distinguish the evidence code used in parent and child term.

The second type is “biological domain-inconsistent annotation.” A GO term should avoid using species-specific definitions and rather include any term that can be applied to more than one taxonomy classes of organisms (The Gene Ontology Consortium, 2000). Some GO terms have species-specific characteristics such as *nucleus* (GO:0005634), specific for eukaryotes and *unidirectional conjugation* (GO:0009291), specific for prokaryotic specific terms. As GO-based annotation expands to various species, however, species-specific terms become increasingly problematic. For example, a gene product having UNIPROT ID O24899 from Helicobacter pylori, a kind of bacteria, is wrongly annotated to *nucleus*, a eukaryote-only GO term.

The third type is “taxonomy inconsistent annotation”. Recently, the GO Consortium provided terms with taxonomy restrictions, containing species-specific terms with the NCBI taxonomy group for which they are or are not appropriate (http://www.geneontology.org/GO.sensu.shtml). Forty four taxonomic groups used taxonomy restricted terms in the January 2010 GO version. Taxonomy inconsistent annotation occurs when a taxonomy restricted term is annotated to a gene that does not belong to the corresponding taxonomy group. GO consortium checks the inconsistent annotation using taxonomy restricted terms and provide reports of inconsistent annotation. But many annotations have been produced without consideration of taxonomy restricted terms. For example, we found that a eukaryote restricted GO term, Golgi apparatus (GO:0005794), was (wrongly) annotated to 27 gene products of *Escherichia coli*, a kind of bacteria.

In the present study, we analyzed the distributions of the semantic inconsistencies in GO-based annotations using 27 major biological databases. To understand the factors influencing such inconsistent annotations, we perform correlation analysis between the inconsistent annotations and the possible attributes for the inconsistent annotations including the usage of evidence codes (http://www.geneontology.org/GO.evidence. shtml), the number of gene products, the number of species and the number of GO terms. We developed a set of web-based utilities, GOChase-II, to correct the semantic inconsistencies in addition to the previous corrections for the syntactic errors by GOChase-I [12].

## Material and methods

### Databases

We obtained GO DB downloads from the GO database site (http://www.geneontology.org/GO.downloads.database.shtml). We collected GO-based annotations for genes and gene products from 27 major biological databases including NCBI’s Gene and Ensembl. The GO DB schema used for data integration was obtained at http://www.geneontology.org/images/diag-godb-er.jpg. To extract GO-update history, we downloaded GO monthly reports from January 2000 to December 2007 from the GO FTP site (ftp://ftp.geneontolgy.org). Since January 2008, GO consortium, however, have not provided monthly reports, thus we use OBO-Edit tool to generate GO change reports over the past month [15]. OBO-Edit generated reports provide four additional types of change; change comment, change synonym, change category, and change external reference. It also provide six types of changes which defined by monthly report; new term, new obsoletion, term name change, new definition, new term merge and term movement. We parsed these 11 types of change for resolving GO-update history. The NCBI taxonomy database (ftp://ftp.ncbi.nih.gov/pub/taxonomy/) was downloaded to find and correct biological domain -inconsistent annotations. The NCBI taxonomy database indexes over 320,000 named organisms that are represented in the databases with at least one nucleotide or protein sequence [16].

### Semantic inconsistencies

The hierarchical relationships extracted from the GO DAGs were used to determine redundant annotations. For each gene product, parent-child relationship between any pair of GO terms annotated to the gene product in the 27 biological databases was tested to determine redundant annotations (Table 1). We analyze the redundant annotation to distinguish between one specific gene product annotated using parent-child terms that use the same evidence code and those use different evidence codes. In some cases (details in introduction section), the redundant annotations of parent-child terms use the different evidence code are supporting data.

**Table 1:** Redundant annotations in biological databases

Databases	**DB version** mm/dd/yy	**GO version**mm/dd/yy	No. of gene products annotated with GO terms			No. of GO annotations applied to gene products			No. of GO terms used in gene product annotations		
			Redundant anntation	Redundant annotation (same evidence code)	Total gene products	Redundant anntation	Redundant annotation (same evidence code)	Total GO annotations	Redundant anntation	Redundant annotation (same evidence code)	Total GO terms

Ensembl^a^	09/01/09	01/01/10	307,467	299,911	673,180	783,687	707,335	4,395,125	2,978	2,551	12,309
Gene^b^	12/15/09	01/01/10	88,797	73,001	235,852	223,772	143,537	1,234,220	3,369	2,632	15,363

AspGD^c^	12/21/09	01/01/10	523	225	3,425	640	259	15,340	239	107	3,259
CGD	11/24/09	01/01/10	474	229	4,040	772	309	20,009	254	104	3,332
dictyBase	12/27/09	01/01/10	2,590	1,619	7,489	4,651	2,377	31,064	368	241	2,403
EcoCyc	12/14/09	01/01/10	173	155	1,869	273	219	4,992	132	111	1,388
FB	10/30/09	01/01/10	3,355	1,823	12,509	7,301	2,740	68,316	1,077	656	4,924
GeneDB_Pfalciparum	10/27/05	01/01/10	21	16	2,206	21	16	4,632	17	15	663
GeneDB_Spombe	09/28/09	01/01/10	2,797	1,330	5,213	4,009	1,662	34,114	495	297	3,394
GeneDB_Tbrucei	07/18/07	01/01/10	234	191	2,977	251	202	10,414	61	52	935
GR_protein	08/26/09	01/01/10	426	369	41,321	552	445	49,721	90	77	646
JCVI_CMR	07/22/09	01/01/10	446	412	21,271	455	417	54,398	90	83	2,350
MGI	12/17/09	01/01/10	14,927	13,466	18,167	50,970	33,966	151,652	1,564	1,214	7,327
NCBI	03/03/08	01/01/10	324	187	11,274	457	319	27,647	66	63	492
PDB	12/17/09	01/01/10	10,234	10,234	21,849	18,263	18,263	83,588	283	283	1,884
PseudoCAP	06/28/06	01/01/10	584	244	1,519	720	275	7,284	54	30	859
RefSeq	12/14/09	01/01/10	1,945	1,945	12,166	2,748	2,748	36,201	125	125	1,440
RGD	10/02/09	01/01/10	9,932	8,008	17,352	29,961	15,120	180,606	1,893	1,341	9,094
SGD	12/25/09	01/01/10	5,273	4,482	6,353	23,815	11,575	76,188	1,118	766	4,222
SGN	10/23/09	01/01/10	12	9	155	12	9	1,253	10	8	653
TAIR	12/23/09	01/01/10	8,102	6,871	51,713	10,615	8,656	149,466	646	473	4,103
TIGR_CMR	11/14/07	01/01/10	757	731	40,653	782	756	101,965	95	92	2,441
UniProt	12/17/09	01/01/10	206	41	1,290	381	67	9,381	11	9	173
UniProtKB/Swiss-Prot	12/17/09	01/01/10	384,061	380,296	419,241	1,303,909	1,279,924	3,416,194	1,709	1,514	11,507
UniProtKB/TrEMBL	12/17/09	01/01/10	3,615,614	3,615,469	5,981,451	9,116,513	9,115,708	28,760,356	1,420	1,402	9,262
WB	11/26/09	01/01/10	5,252	5,041	15,667	9,904	8,926	91,611	497	381	2,738
ZFIN	12/23/09	01/01/10	7,047	6,856	15,074	17,683	16,454	101,152	603	509	3,019

a http://www.ensembl.org/index.html

b http://www.ncbi.nlm.nih.gov/gene

c http://www.geneontology.org/GO.downloads.annotations.shtml

To find biological domain inconsistency in GO annotation, we reviewed and manually extracted 410 ‘eukaryote-only’ and 73 ‘prokaryote-only’ GO terms including such terms as *RNA import into nucleus* and *ketodeoxyoctanoate biosynthesis* (see additional file 1 and 2). All gene products in the 27 databases were divided into non-prokaryotic and non-eukaryotic classes according to the species definition in NCBI taxonomy. Biological-domain-inconsistent annotation was determined by testing the consistency between the corresponding species of a gene product and the ‘prokaryote-only’ or ‘eukaryote-only’ classification of the annotation term.

There were 44 taxonomy groups having taxonomy restricted terms in the January 2010 GO version. The taxonomy inconsistent annotation was determined by inconsistency between species-specific GO terms and the species of origin of the annotated gene products.

### Attributes for inconsistent annotation

In search for the possible attributes for the inconsistent annotations, we evaluated five possible attributes by correlation analysis; the use of different evidence codes, the number of gene products, the number of species, the number of GO terms, and the average number of GO annotations. Every GO annotation is supposed to indicate the type of evidence. There are 18 evidence codes currently available. When no evidence code was assigned for an annotation, we marked it as ‘Not Available (NA)’.

## Results

To analyze the distributions of the semantic inconsistencies in GO-based annotations we calculated the distribution of redundant annotations in the 27 biological databases (Table 1). All databases have redundant annotation. The fraction of redundant annotations in databases is distributed from 0.9% to 91% for gene products (31% in average), from 2% to 26% for GO terms (13% in average), and from 0.4% to 38% for GO annotations (12% in average). UniProtKB/Swiss-Prot shows the highest redundancy for gene product (91%) and GO annotation (38%). The database showing the highest redundancy in GO terms is Ensembl (24%). GeneDB_Pfalciparum shows the lowest numbers among the databases; 0.9% for gene products, 2.5% for GO terms and 0.4% for GO annotations. In all databases, the fractions of redundant annotation based on the same evidence code are larger than different evidence code.

The distributions of biological-domain-inconsistent annotations are calculated using prokaryote-only and eukaryote-only GO terms we defined. Biological domain inconsistent annotation was found in thirteen databases of non-prokaryotic gene product and eight databases of non-eukaryotic gene product (Table 2). Most of databases have less than 100 inconsistent annotations, except four databases (Ensembl, NCBI Gene, UniProtKB/Swiss-Prot and UniProtKB/TrEMBL). In both biological domains, UniProtKB/TrEMBL is shown to have the highest portion of inconsistent annotation by all three measures. Taxonomy inconsistent annotations were found in 27 out of the 44 taxonomy groups having at least one taxonomy restricted GO term (Table 3 in additional file 3, see method). Table 3 in Additional file 3 shows the numbers of taxonomy inconsistent annotations (as numerators) and the numbers of taxonomy restricted GO terms used (as denominators) in the 27 databases. A blank cell means no annotation with taxonomy restricted GO term. Taxonomy inconsistent annotations are not evenly distributed across databases or taxonomy groups (Table 3 in additional file 3). The NCBI Gene, Ensemble, UniProtKB/Swiss-Prot and UniProtKB/TrEMBL, for example, has inconsistent annotation in most of taxonomy groups as while, no taxonomy inconsistent annotation was found in the five databases: CGD (0/2174), GeneDB_Tbrucei (0/422), NCBI (0/1291), PseudoCAP(0/20), and UniProt (0/17). Interestingly, all annotations for *Passeriformes* (11/11) are taxonomy inconsistent. *Cellular organisms* show the lowest taxonomy-inconsistent annotations rate (7/42344) among the 27 taxonomy groups.

**Table 2:** Biological-domain-inconsistent annotations in biological databases

Biological Domain	Databases	**DB version** mm/dd/yy	**GO version** mm/dd/yy	No. of gene products annotated with GO terms		No. of GO annotations applied to gene products		No. of GO terms used in gene product annotations	
				Biological-domain inconsistent	Total gene product	Biological-domain inconsistent	Total GO annotation	Biological-domain inconsistent	Total GO terms

Non-prokaryotic gene product	Ensembl^a^	09/01/09	01/01/10	711	1,891,586	760	4,395,125	13	12,309
	Gene^b^	12/15/09	01/01/10	1,517	2,391,443	1,647	1,133,060	34	14,762

	AspGD^c^	12/21/09	01/01/10	2	3,425	2	15,340	1	3,259
	dictyBase	12/27/09	01/01/10	1	7,489	1	31,064	1	2,403
	FB	10/30/09	01/01/10	1	12,509	1	68,316	1	4,924
	GeneDB_Tbrucei	07/18/07	01/01/10	2	2,977	2	10,414	1	935
	MGI	12/17/09	01/01/10	2	18,167	2	151,652	2	7,327
	PDB	12/17/09	01/01/10	26	9,170	26	31,686	3	1,024
	RGD	10/02/09	01/01/10	2	18,363	4	180,606	3	9,094
	TAIR	12/23/09	01/01/10	3	51,713	3	149,466	1	4,103
	UniProtKB/Swiss-Prot	12/17/09	01/01/10	2,680	122,261	3,803	1,035,209	17	10,589
	UniProtKB/TrEMBL	12/17/09	01/01/10	20,573	2,333,592	23,876	12,234,060	24	8,586
	WB	11/26/09	01/01/10	12	15,667	13	91,611	5	2,738

Non-eukaryotic gene product	Geneb^b^	12/15/09	01/01/10	53,088	3,595,041	76,597	101,160	319	2,497
	
	EcoCyc^c^	12/14/09	01/01/10	2	1,869	2	4,992	1	1,388
	JCVI_CMR	07/22/09	01/01/10	16	21,271	16	54,398	3	2,350
	PDB	12/17/09	01/01/10	83	16,580	85	66,027	12	1,689
	TIGR_CMR	11/14/07	01/01/10	70	40,653	70	101,965	4	2,441
	UniProt	12/17/09	01/01/10	48	248	67	7,870	3	44
	UniProtKB/Swiss-Prot	12/17/09	01/01/10	4,454	324,523	5,297	2,581,774	30	3,306
	UniProtKB/TrEMBL	12/17/09	01/01/10	77,047	4,459,834	83,965	20,009,318	49	4,048

a http://www.ensembl.org/index.html

b http://www.ncbi.nlm.nih.gov/gene

c http://www.geneontology.org/GO.current.annotations.shtml

To investigate which factors are related to each inconsistent annotation we analyzed correlation between three types of inconsistent annotation and 23 possible attributes of inconsistent annotation (Table 3). As shown in table 3, Inferred from Electronic Annotation (IEA) shows the highest correlation with redundant (r=0.99) and taxonomy inconsistent annotation (r=0.99). Biological domain inconsistent annotation shows high correlation with number of gene product (0.97). We found that the numbers of species and average number of GO annotation show high correlation while the number of GO term shows low correlation with all types of inconsistent annotation (Table 3).

**Table 3:** Gene Ontology distribution incorrectly annotated across evidence codes and the related factors

Evidence code	No. of inaccurate annotation (correlation coefficient value)				Total No. of GO annotation
		
	Redundant annotation	Biological domain inconsistent annotation	Taxonomy inconsistent annotation	Total inaccurate annotation	
NR	0 (*)	0 (*)	0 (*)	0 (*)	6
ISM	30 (-0.07)	2 (0.26)	0 (*)	32 (-0.06)	279
ISA	287 (-0.05)	1,385 (0.40)	0 (*)	1,672 (-0.04)	11,756
IGC	322 (-0.05)	11 (0.43)	0 (*)	333 (-0.04)	888
IC	1,193 (-0.03)	1,265 (0.40)	0 (*)	2,458 (-0.02)	12,490
IEP	2,344 (-0.03)	2,467 (0.46)	0 (*)	4,811 (-0.02)	27,889
EXP	3,273 (0.08)	1,221 (0.16)	0 (*)	4,494 (0.08)	20,781
IGI	3,628 (-0.02)	4,171 (0.41)	0 (*)	7,799 (-0.02)	34,985
RCA	6,469 (-0.07)	7,710 (0.18)	0 (*)	14,179 (-0.06)	85,014
NAS	7,921 (0.01)	4,285 (0.39)	0 (*)	12,206 (0.02)	58,687
IPI	10,555 (0.11)	1,163 (0.31)	0 (*)	11,718 (0.11)	72,597
ISO	14,119 (-0.05)	15,956 (0.39)	16 (-0.06)	30,091 (-0.04)	115,268
TAS	15,944 (0.01)	8,331 (0.39)	5 (-0.01)	24,280 (0.01)	113,414
ND	18,987 (-0.07)	1 (0.51)	0 (*)	18,988 (-0.05)	366,152
ISS	24,314 (0.01)	12,828 (0.53)	49 (0.01)	37,191 (0.03)	377,770
IMP	25,994 (-0.03)	29,932 (0.38)	160 (-0.06)	56,086 (-0.03)	242,825
IDA	44,327 (0.01)	29,863 (0.38)	17 (-0.02)	74,207 (0.01)	311,481
IEA	11,433,355 (0.99)	219,560 (0.75)	56,180 (0.99)	11,709,095 (0.99)	42,984,075
NA (Not Avaliable)	803 (-0.02)	3,654 (0.61)	12 (-0.04)	4,469 (-0.01)	18,102

No of gene product	(0.71)	(0.97)	(0.69)	(0.72)	
No. of species	(0.99)	(0.78)	(0.99)	(0.99)	
No. of GO term	(0.35)	(0.57)	(0.34)	(0.36)	
Average No. of annotations	(0.99)	(0.76)	(0.99)	(0.99)	

### GOChase-II implementation

GOChase-I [12] is a set of web-based utilities to detect and correct *syntactic* errors from GO-based annotations caused by GO versioning and tracing problems. On the contrary, GOChase-II (http://www.snubi.org/software/GOChase2/) attempts to correct *semantic* errors in GO-based annotations. It provides four web-based interfaces. (1) GOChase-History resolves the whole evolution history of a GO ID. As an example, the GO term, *sorocarp development* (GO:0030587), has repeatedly swung back and forth among the fifteen GO terms (*reproduction*, *cell communication*, *development*, *response to external stimulus*, *physiological process*, *biological_process*, *response to biotic stimulus*, *morphogenesis*, *multicellular organismal development*, *anatomical structure morphogenesis*, *anatomical structure development*, *asexual reproduction*, *fruiting body development in response to starvation*, *fruiting body development*, *response to starvation*) by the 31 GO operations in fifteen updates between March 2002 and November 2008. (2) GOChase-Species resolves the distribution of the usage of a GO term across different species and displays the distribution onto the taxonomy tree. The Species function is a powerful tool to analyze the species specificity of a GO term. Some terms are limited to specific species whereas others are used for a wide variety of species. For example, *negative regulation of vulval development* (GO:0040027) is annotated 395 times but exclusively to *Caenorhabditis elegans* (i.e. 100%). It is suggested that *cyanelle* may be a species-specific term. We identified 3548 GO terms annotated only to a single species in January 2010 GO version (see additional file 4). On the other hand, *oxidoreductase activity* (GO:0016491) is annotated 800,048 times to 108,929 different species (i.e. 7.3 times per a species in average). Species function can also be used to find the wrong use of species-specific terms. (3) GOChase-Correct highlights a 'merged-term' and redirects it to the correct 'target term' into which the terms have been merged. For an obsolete term, GOChase provides the alternative terms. GOChase-Correct correct redundant and biological-domain-inconsistent annotations. (4) When one inputs a GO ID, GOChase will resolve all gene products annotated with the GO ID across all the databases in Table 1. GOChaser provides GO enrichment analysis for input gene-expression clusters. Although most GO enrichment analysis tools have the similar functionality [14], GOChaser has a unique functionality of correcting both the syntactic and semantic errors to improve the analysis results. GOChaser provides two statistical models, the hypergeomeric test and the Fisher’s exact test, with multiple hypotheses testing correction (Bonferroni correction).

## Conclusion and discussion

We identified and corrected three types of semantic inconsistencies in GO-based annotations for gene products from 27 major biological databases. GO becomes a widely accepted ontology in biomedical field. The under-managed errors and inconsistencies may reflect its short history, its ever growing complexity, and the vast amount of the biological domain knowledge. Recently GO Consortium starts working on refining GO contents and structure [17]. The present study demonstrates that the GO community may be empowered by bioinformatics tools ensuring error-proof mechanisms concerning the GO hierarchical relationships, species-specific definitions and GO term usage guidelines.

To sum up our result in this research, there is no database free from the semantic inconsistent annotation. Among the three types of semantic inconsistent annotation, redundant annotation is most common error. About 12% of the whole annotations are redundant. Only a few biological-domain inconsistent annotations are found in the 18 biological databases because of the small number of ‘eukaryote-only’ (410) and ‘prokaryote-only’ (71) GO term.

The high correlation between IEA and inconsistent annotations (Table 3) suggests that IEA has lower reliability than others. Electronically generated associations without human judgment are labelled as IEA. GO Consortium proposes a hierarchy of reliability among evidence codes (http://www.geneontology.org/GO.evidence.shtml). In general, TAS and IDA show higher reliability. TAS and IDA have low correlation with all three types of inconsistent annotation. And most of evidence codes, which curated by human, have low correlation with all types of inconsistent annotation. This result implies that the hierarchy of reliability among evidence codes are preserved in inaccurate annotation.

The numbers of gene products and species of a database show high correlations with all types of inconsistent annotations except taxonomy-inconsistent annotation. It suggests that the complexity of database maintenance may affect the occurrence of inconsistent annotations. Therefore, it is more strongly required for such databases to implement a sound mechanism such as GOChase-II in order to avoid semantic inconsistencies caused by multiple user-groups.

## Authors' contributions

YRP conceived the study, wrote the manuscript and implemented the web-based program. JK wrote the manuscript and validated the inconsistent annotation. HWL validated the taxonomy-specific GO terms and helped to draft the manuscript. YJY calculated history data of GO term. JHK coordinated and supervised the study. All authors read and approved the final manuscript.

## Supplementary Material

Additional file 1 Additional file 1 Click here for file

Additional file 2 Additional file 2 Click here for file

Additional file 3 Additional file 3 Click here for file

Additional file 4 Additional file 4 Click here for file
