# Supplementary material for: GOChase-II: correcting semantic inconsistencies from Gene Ontology-based annotations for gene products
Source: BMC Bioinformatics. 2011 Feb 15;12(Suppl 1):S40. doi: 10.1186/1471-2105-12-S1-S40 (PMC3044297; doi:10.1186/1471-2105-12-S1-S40)
Supplement: Additional file 3 [file 1471-2105-12-S1-S40-S3.pdf]

Table 3 – *Taxon*-inconsistent annotation in Gene Ontology-based annotations for gene products in selected databases

Database	DB version mm/dd/yy	GO version mm/dd/yy	Apicomplexa	Arthropoda	Ascomycota	Aves	Bacteria	Bilateria	cellular organisms	Chordata	Deuterostomia	Eukaryota	Fungi
Ensembl[a]	09/01/09	01/01/10		45/413	0/91	13/349	495/495		0/9348	74/1594	41/841	0/213861	35/553
Gene[b]	12/15/09	01/01/10	0/468	30/728	11/298	0/35	9/1232	1/2211	0/4653	0/194	0/119	1/54566	24/2735
AspGD[c]	12/21/09	01/01/10			0/39		2/2		0/57			0/945	0/265
CGD	11/24/09	01/01/10			0/41				0/359			0/1227	0/547
dictyBase	12/27/09	01/01/10					1/1	1/1	0/38			0/816	4/4
EcoCyc	12/14/09	01/01/10					0/16		0/49			2/2	
FB	10/30/09	01/01/10		0/861			1/1		0/221			0/2399	
GeneDB_Pfalciparum	10/27/05	01/01/10	0/553						0/59			0/74	
GeneDB_Spombe	09/28/09	01/01/10			0/103				0/269			0/4536	0/417
GeneDB_Tbrucei	07/18/07	01/01/10							0/74			0/348	
GR_protein	08/26/09	01/01/10							0/13			0/11006	
JCVI_CMR	07/22/09	01/01/10					0/310		0/395			2/2	
MGI	12/17/09	01/01/10		2/2		0/6			0/239	0/49	0/32	0/8263	2/2
NCBI	03/03/08	01/01/10			0/47				0/3			0/172	0/1069
PDB	12/17/09	01/01/10					4/190		0/34			19/126	
PseudoCAP	06/28/06	01/01/10					0/11						
RefSeq	12/14/09	01/01/10		1/1					0/12			0/792	
RGD	10/02/09	01/01/10		2/2		0/16			0/530	0/73	0/39	0/8512	7/7
SGD	12/25/09	01/01/10			0/109				0/210			0/7038	0/815
SGN	10/23/09	01/01/10							0/3			0/52	1/1
TAIR	12/23/09	01/01/10					3/3		0/562			0/4772	
TIGR_CMR	11/14/07	01/01/10					0/448		0/139			1/1	
UniProt	12/17/09	01/01/10										0/15	
UniProtKB/Swiss-Prot	12/17/09	01/01/10	0/36	4/62		0/14	957/13765	0/12	0/3290	0/90	0/58	1949/48001	56/161
UniProtKB/TrEMBL	12/17/09	01/01/10	1/301	39/109		0/3	5699/75122		7/16255	0/156	0/34	18338/432429	781/1161
WB	11/26/09	01/01/10		20/20			2/2	0/7338	0/5485			0/1229	
ZFIN	12/23/09	01/01/10				0/1			0/47	0/4	0/22	0/3412	
Total taxonomy inconsistent annotation			1/1358	143/2198	11/728	13/424	7173/91598	2/9562	7/42344	74/2160	41/1145	20312/804596	910/7737

Database	Fungi and Ascomycota	Glaucocystophyceae	Insecta	Magnoliophyta	Mammalia	Mammalia or Theria	Metazoa	Passeriformes	Prokaryota	Protostomia	Theria	Vertebrata	Viridiplantae	Viridiplantae or Bacteria or Euglenozoa	Viridiplantae or Bacteria or Euglenozoa or Alveolata	Viridiplantae or Euglenozoa	Total taxonomy inconsistent annotation	Total GO annotation
Ensembl[a]	0/1297		0/184	62/62	279/2748	724/6128	574/11871	7/7	32/34	0/110	174/1330	11/74	19/22	47/50	144/157	1/1	2777/251620	4,395,125
Gene[b]	3/224		0/305	0/162	30/474	44/589	6/5926	2/2	2/49	64/210	17/178	0/20	48/1188	2/284	9/77	3/2924	306/79851	1,234,220
AspGD[c]																	2/1308	15,340
CGD																	0/2174	20,009
dictyBase	0/2					1/1	3/3						1/1				11/867	31,064
EcoCyc									0/1								2/68	4,992
FB	0/2		0/362				0/527			0/249							1/4622	68,316
GeneDB_Pfalciparum	0/1						1/1						6/6				7/694	4,632
GeneDB_Spombe	1/1																1/5326	34,114
GeneDB_Tbrucei																	0/422	10,414
GR_protein			1/1	0/21									0/27		0/5		1/11073	49,721
JCVI_CMR									0/28					0/1	0/1		2/737	54,398
MGI	0/40				0/100	0/205	0/633	1/1			0/42	0/1					5/9615	151,652
NCBI																	0/1291	27,647
PDB							20/25		0/93				0/6	0/129	10/100	1/11	54/714	83,588
PseudoCAP									0/5					0/4			0/20	7,284
RefSeq	0/1						0/61										1/867	36,201
RGD	0/102				0/209	0/216	0/609	1/1	1/1		0/63	0/2	3/3	1/1			15/10386	180,606
SGD	2/2												21/21				23/8195	76,188
SGN					1/1	1/1	3/3				1/1		0/3		0/2	0/1	7/68	1,253
TAIR				0/180									0/1307	0/424	0/88	0/3602	3/10938	149,466
TIGR_CMR									0/46				1/1	0/2			2/637	101,965
UniProt													0/1			0/1	0/17	9,381
UniProtKB/Swiss-Prot	8/140	4/243	0/43		11/309	24/224	58/2221		48/663		7/70	0/1	40/317	1150/15314	700/6664	2880/19353	7896/111051	3,416,194
UniProtKB/TrEMBL	0/151	96/101	0/47		52/213	37/269	921/14173		1540/7912	0/7	14/105	0/3	40/2087	2818/30094	4608/42301	10234/135168	43225/758401	28,760,356
WB	0/2						0/9436		1/1	73/73				1/1	9/9		106/23596	91,611
ZFIN	0/2				1/1		0/150					0/21					1/3660	101,152
Total taxonomy inconsistent annotation	14/1967	100/344	1/942	62/425	374/4055	831/7633	1586/45639	11/11	1624/8833	137/649	213/1789	11/122	179/4990	4019/46304	5480/49604	13119/161061	56448/1298218	39,116,889

a http://www.ensembl.org/index.html

b http://www.ncbi.nlm.nih.gov/gene

c http://www.geneontology.org/GO.current.annotations.shtml
